# The Prevalence of Depression and Anxiety Symptoms and Their Association With Respiratory Diseases

**DOI:** 10.7759/cureus.49488

**Published:** 2023-11-27

**Authors:** Alejandro Hernández-Solís, Arturo Reding-Bernal, Pablo Álvarez-Maldonado, Eliasib Mojica Jaimes, Fryda Jareth Serna Valle, Andrea Quintana Martínez, Merari Velazquez Gachuz

**Affiliations:** 1 Pulmonology and Thorax Surgery Service, “Dr. Eduardo Liceaga” General Hospital of Mexico, Mexico, MEX; 2 Research, “Dr. Eduardo Liceaga” General Hospital of Mexico, Mexico, MEX; 3 Surgery, Anahuac University Mexico North Campus, Mexico, MEX

**Keywords:** comorbidities, hospital anxiety and depression scale, depression, anxiety, respiratory diseases

## Abstract

Introduction: Patients with respiratory diseases face adverse situations such as symptom management, general condition deterioration, and a hostile perception of the hospital environment, favoring the appearance of anxiety and depression.

Methods: A total of 317 patients hospitalized for a disease of pulmonary origin were analyzed and divided into the following subgroups: infectious, oncological, acute, and chronic diseases. Patients over 18 years of age with preserved cognitive capacity were included in the study. The Hospital Anxiety and Depression Scale (HADS) was applied to them on the second or fourth day of their hospital stay and five days after the first evaluation. Multiple linear regression models were carried out to analyze the association between anxiety and depression measured over two different periods. The models present the statistically significant variables with a 95% confidence level.

Results: The patients presented with anxiety in 74.4% of cases, mainly those with acute respiratory diseases (42.4%) and neoplastic diseases (27.5%). A total of 69.5% presented with depression, with symptoms more significant in those with chronic and oncological pulmonary diseases and those with no job. Patients with at least one comorbidity presented with anxiety in 53.9% of cases and depression in 52.1% of cases. Linear regression models were carried out and showed that anxiety was 1.75 and 1.84 times more frequent in patients with chronic diseases compared to those with infectious pathologies in the first and second reviews, respectively. The linear regression model also showed a higher frequency of depressive symptoms in patients with chronic conditions (1.62 times) compared to the group with infectious and contagious pathologies, and prolonged hospital stays were associated with depressive symptoms 1.37 times more than short stays.

Conclusions: Anxiety and depression are frequent disorders in patients with respiratory diseases, negatively affecting the prognosis. Routine mental health screening and multidisciplinary management are essential in this population.

## Introduction

In Mexico, respiratory diseases are among the 10 most common causes of death. Infectious diseases such as influenza and pneumonia increase with age, while chronic diseases are the leading cause of death from age 55; among them are bronchial asthma, chronic obstructive pulmonary disease (COPD), obstructive sleep apnea (OSA) syndrome, and lung cancer. However, after the SARS-CoV-2 pandemic, respiratory diseases became the leading cause of death [1,2]. In European countries, for example, an increase of 68.5% in deaths from respiratory diseases was observed in 2020 compared to 2019 [3].

In people suffering from respiratory diseases, comorbidities often worsen exacerbations, leading to a longer hospital stay and impaired quality of life. Among these comorbidities, psychiatric diseases such as depression and anxiety stand out, which are frequent and are shown to impact the prognosis and outcomes. For each hospital admission, the symptoms of anxiety and depression worsen, and consequently, the exacerbations do as well, perpetuating a vicious circle, and factors such as poor adherence to treatment or abandonment of it could be related to these psychiatric disorders. Knowing the prevalence of anxiety and depression in this population is the first step to generating health policies that favor the comprehensive management of these patients and thus break the vicious cycle that perpetuates these symptoms [4].

Depression is a disorder characterized by sadness, a lack of interest in previously satisfying activities, disturbances in sleep and appetite patterns, unusual fatigue, and an inability to concentrate, causing increased stress. Generalized anxiety disorder is defined as excessive and permanent worry lasting at least six months [5].

In 2019, it was estimated that 280 million people suffered from depression, with those over 60 years of age being the most affected, as a result of the COVID-19 pandemic. In 2020, an increase in mortality was recorded in patients with respiratory diseases, reaching rates of up to 68.5% compared to the previous year in countries such as Spain [4-6]. The Global Burden of Disease estimated that with the COVID-19 pandemic, cases of major depressive disorder increased by 27.6% while anxiety disorders increased by 25.6% globally [7]. In Mexico, the estimated prevalence of depression was 16.1% in the adult population in 2021, which was more common in females. Regarding anxiety disorders, the global prevalence is between 4% and 24%, and in the Mexican population, it is 19.3% for severe anxiety and 31.3% for moderate anxiety [8-10]. People with respiratory diseases show higher rates of anxiety and depression than the rest of the population, which can be explained by the emotional burden and characteristic symptoms of conditions such as dyspnea [4,11,12].

It is vital to understand dyspnea as a two-dimensional phenomenon, both physical and emotional. Symptoms such as tachycardia, chest tightness, and dysfunctional breathing are part of the clinical context of both respiratory and psychiatric illnesses, leading to anxiety and depression being underdiagnosed in patients with respiratory disorders [4,12].

This in turn leads to an increase in interleukin (IL) 1β, IL-8, IL-6, tumor necrosis factor-alpha (TNF-α), interferon-γ, C-X-C motif chemokine ligand 10 (CXCL10), and chemokine such as chemokine (C-C motif) ligand 2 (CCL2), which activate the T-helper lymphocytes. Consequently, neuronal inflammation is triggered, along with the dysfunction of the hypothalamic-pituitary-adrenal axis. Additionally, the induction of indoleamine 2,3-dehydrogenase occurs, resulting in the reduced production of serotonin and the increased production of kynurenic and quinolonic acids. The former two stimulate the release of glutamate, decreasing trophic factors related to depression [4,11,13,14]. Therefore, it is not strange to think that the rates of anxiety and depression in this population are higher than those considered and that they are underdiagnosed.

The objective of this study is to identify the prevalence of anxiety and depression in patients hospitalized in a respiratory care unit in Mexico City, as well as the factors associated with these conditions.

## Materials and methods

This prospective, longitudinal, analytical study was conducted from April 2019 to December 2020. Three hundred seventeen patients hospitalized at the “Dr. Eduardo Liceaga” General Hospital of Mexico, a tertiary public teaching hospital in Mexico City, for a disease of pulmonary origin were included and divided into the following subgroups: infectious, oncological, acute, or chronic disease. The sample was selected based on the inclusion, exclusion, and elimination criteria. The inclusion criteria include patients older than 18 years with preserved cognitive capacity and two or more days of hospitalization. The exclusion criteria include patients already in psychiatric treatment. The elimination criteria include patients unable to complete the survey in its entirety and those who died or were discharged from the hospital before the second evaluation.

Sociodemographic factors, the presence of chronic degenerative diseases, the reason for admission to the hospital, nutritional status, the supplementary use of oxygen, and days of hospital stay were analyzed.

The Hospital Anxiety and Depression Scale (HADS) was applied to the participants [15,16]. This scale is a 14-item questionnaire composed of two subscales, one for anxiety (Hospital Anxiety and Depression-Anxiety {HADA}) and another for depression (Hospital Anxiety and Depression-Depression {HADD}); each subscale consists of seven items on a Likert scale from 0 to 3, giving a minimum score of 0 and maximum of 21 for each subscale. Cutoff points from 0 to 7 imply the absence of anxiety and depression or symptoms without clinical relevance, 8-10 points are for symptoms to be considered probable cases, and 11-21 points are for symptoms with clinical relevance that require attention. This is a validated instrument for screening hospitalized patients with chronic conditions that assesses cognitive and behavioral symptoms and is easy to understand and apply, for both healthcare personnel and hospitalized patients. The instrument was applied between the second and fourth days of the hospital stay and five days after the first evaluation.

Multiple linear regression models were performed to analyze the association between anxiety and depression measured over two different periods. The variables with which their association was analyzed were age, gender, respiratory diseases (acute, chronic, infectious, or oncological), occupation, socioeconomic level (according to the Mexican Association of Market Research Agencies), hospital stay, the requirement of supplemental oxygen, comorbidities, the place of origin, and nutritional status. The models present the variables found to be statistically significant with a confidence level of 95%. The statistical software used for the analysis was Stata version 15.1 (StataCorp LLC, College Station, TX).

Each patient was informed of the purpose of the study, and each of them signed an informed consent form; likewise, the data management was for research purposes only and confidentially, and this study had the approval of the Research Ethics Committee, Research Committee, and Biosafety Committee of the “Dr. Eduardo Liceaga” General Hospital of Mexico (approval number: DI/20/503/04/44).

## Results

A total of 317 patients were included, of which 181 (57.1%) were males and 136 (42.9%) were females. Respiratory diseases were divided into four groups: 1) infectious-contagious illness (149, 47.0%), including COVID-19 (80), tuberculosis (35), bacterial pneumonia (20), and empyema (14); 2) oncological disease (80, 25.2%), including lung cancer (68) and metastatic pleural effusion (12); 3) chronic disease (50, 15.7%), including asthma (30), COPD (10), and OSA (10); and 4) acute disease (38, 11.9%), including pulmonary thromboembolism (29) and chest trauma (9).

Regarding the socioeconomic level, the majority belonged to an extremely very low level (110, 34.8%), followed by an extremely low level (101, 32.0%) and a low level (80, 25.3%). The predominant place of origin was urban (223, 70.6%). At the time of the study, 52.5% of patients had no job. Associated comorbidities were diabetes mellitus in 92 (29.0%), systemic arterial hypertension (SAH) in 86 (27.1%), chronic cardiovascular disease (CCD) in 31 (9.8%), and chronic kidney disease in 30 (9.5%). Of the patients, 160 (50.5%) were oxygen-dependent (Table 1).

**Table 1:** Sociodemographic and Clinical Characteristics of Patients Hospitalized in a Respiratory Care Unit

Variables	Total		Infectious		Chronic		Acute		Neoplasia	
	n	%	n	%	n	%	n	%	n	%
Sex										
Male	181	57.1	99	66.4	21	42.0	24	63.2	37	46.3
Female	136	42.9	50	33.6	29	58.0	14	36.8	43	53.8
Socioeconomic level										
C+	3	1.0	2	1.4	-	-	-	-	1	1.3
C	22	7.0	10	6.8	3	6.0	-	-	9	11.3
D+	80	25.3	38	25.7	11	22.0	11	29.0	20	25.0
D	110	34.8	52	35.1	19	38.0	13	34.2	26	32.5
E	101	32.0	46	31.1	17	34.0	14	36.8	24	30.0
Origin										
Rural	93	29.4	43	28.9	16	32.0	9	23.7	25	31.7
Urban	223	70.6	106	71.1	34	68.0	29	76.3	54	68.4
Occupation										
Employee	150	47.5	67	45.0	19	38.0	17	46.0	47	58.8
Unemployed	166	52.5	82	55.0	31	62.0	20	54.1	33	41.3
Diabetes mellitus										
No	225	70.98	103	69.13	34	68	33	86.84	55	68.75
Yes	92	29.02	46	30.87	16	32	5	13.16	25	31.25
Arterial hypertension										
No	231	72.87	117	78.52	22	44	32	84.21	60	75
Yes	86	27.13	32	21.48	28	56	6	15.79	20	25
Oxygen dependence										
No	157	49.5	83	55.7	15	30.0	21	55.3	38	47.5
Yes	160	50.5	66	44.3	35	70.0	17	44.7	42	52.5

During the first evaluation, 236 patients (74.4%) had anxiety symptoms, of which 108 (45.8%) were classified within the category “symptoms without clinical relevance,” being the predominant form; 93 patients (39.4%) entered the category “with symptoms to be considered as a probable case”; and 35 patients (14.8%) presented clinically relevant symptoms that required attention. The highest number of cases of anxiety was found among patients with acute respiratory disease, with 100 cases (42.4%), and neoplastic disease, with 65 cases (27.5%) (Table 2).

**Table 2:** Sociodemographic Characteristics According to Whether Anxiety or Depression Is Present *The comorbidities were diabetes mellitus, arterial hypertension, chronic cardiac disease, and chronic kidney disease

Variables	Anxiety		Depression	
	Measurement 1	Measurement 2	Measurement 1	Measurement 2
	n (%)	n (%)	n (%)	n (%)
Sex				
Female	53 (41.4)	59 (41.0)	55 (48.2)	61 (47.7)
Male	75 (58.6)	85 (59.0)	59 (51.8)	67 (52.3)
Occupation				
Employee	69 (54.3)	77 (53.8)	54 (47.8)	63 (49.6)
Unemployed	58 (45.7)	66 (46.2)	59 (52.2)	64 (50.4)
Socioeconomic level				
Medium	9 (7.0)	12 (8.3)	10 (8.8)	13 (10.1)
Low	119 (93.0)	132 (91.7)	104 (91.2)	115 (89.9)
Comorbidities*				
None	69 (53.9)	75 (52.1)	62 (54.4)	68 (53.1)
At least one	59 (46.1)	69 (47.9)	52 (45.6)	60 (46.9)

The linear regression model shows the association between anxiety and prolonged hospital stays in the group of patients with chronic diseases. Regarding the symptoms of anxiety disorder, people with chronic diseases presented 1.75 and 1.84 times more compared to people with infectious diseases in the first and second measurements, respectively; people with acute respiratory diseases in the first evaluation turned out to be statistically significant (p-value < 0.05) but not in the second measurement (Table 3).

**Table 3:** Multiple Linear Regression Models for Assessing the Association With Anxiety Measured at Two Different Periods LOHS: length of hospital stay

First measurement anxiety model			Second measurement anxiety model		
	Coefficient	P-value		Coefficient	P-value
Age	-0.01	0.180	Age	-0.02	0.046
Sex			Sex		
Female	0.01	0.964	Female	0.21	0.546
Illness group			Illness group		
Infectious	Reference group	-	Infectious	Reference group	-
Chronic	1.75	0.002	Chronic	1.84	0.001
Acute	1.32	0.032	Acute	0.85	0.136
Neoplasia	0.85	0.073	Neoplasia	0.68	0.121
LOHS ≥ nine days	1.63	<0.001	LOHS ≥ nine days	1.55	<0.001

A total of 220 patients (69.5%) presented depressive symptoms, with the category “symptoms without clinical relevance” being the most frequent with 106 (48.2%), “symptoms to be considered as a probable case” with 88 (40.0%), and relevant symptoms that require attention with 26 (11.8%) (Table 2).

Chronic respiratory and oncological diseases were the group of diseases with the most symptoms. In the linear regression model, the association of variables shows that, in the first evaluation of depression, gender and age did not turn out to be statistically significant. At the same time, depressive symptoms were 1.62 times more frequent in patients with chronic diseases compared to patients with infectious diseases; this is in comparison with people with infectious diseases (reference group).

Patients with extended stays (nine days or more) presented depressive symptoms 1.37 times more than those with short stays (less than nine days). In the second sample, in the groups of chronic diseases and neoplasms, depressive symptoms increased, showing a frequency of 1.47 and 1.32 on average, respectively, compared to people with infectious diseases. The depressive symptoms were maintained in the two chronic respiratory and neoplastic disease evaluations (Table 4).

**Table 4:** Multiple Linear Regression Model for Assessing the Association With Depression Measured at Two Different Periods LOHS: length of hospital stay

First measurement depression model			Second measurement depression model		
	Coefficient	P-value		Coefficient	P-value
Age	0.00	0.823	Age	0.11	0.317
Sex			Sex		
Female	0.27	0.475	Female	0.38	0.292
Illness group			Illness group		
Infectious	Reference group	-	Infectious	Reference group	-
Chronic	1.62	0.004	Chronic	1.47	0.005
Acute	-0.14	0.817	Acute	-0.23	0.687
Neoplasia	1.76	<0.001	Neoplasia	1.32	0.003
LOHS ≥ nine days	1.37	<0.001	LOHS ≥ nine days	1.43	<0.001

## Discussion

Depression and anxiety in hospitalized patients make it difficult to adhere to treatment, delay recovery, increase the risk of mortality and the severity of exacerbations, increase hospital stays, and increase care costs. There is a mutual causality since mental illnesses can be a risk factor for suffering from a chronic illness and vice versa. Treatment based on self-management, pulmonary rehabilitation, and cognitive behavioral therapy is shown to be beneficial in the long term [11,12,14].

The Global Burden of Disease reported after the first year of the COVID-19 pandemic that major depressive disorder and anxiety disorder showed a worldwide increase of 25.6% in 2020, with greater involvement in females, young people, and adults. In our series, 75 (93.7%) of the patients with COVID-19 presented severe symptoms of anxiety and depression [7]. Contrary to what has been reported in the literature that psychiatric disorders affect females more, in our study, the male gender (58.59% and 59.03%) registered higher anxiety levels compared to the female gender (41.41% and 40.97%) in both measurements [4,13,17,18].

Socioeconomic status influences depression and anxiety derived from not having social security, medications, and adequate nutrition. In our series, a large part of the population belonged to a medium-low socioeconomic level, increasing the symptoms of anxiety and depression due to the lack of economic resources [11,12,19,20].

In urban areas, depression is the most common disorder in adults, with females being the most affected. This is explained by psychosocial factors such as insecurity, pollution, and accidents, causing permanent stress that results in depression, anxiety, and suicidal ideation. Seventy percent of our studied population lives in an urban environment, and 29.4% comes from a rural environment, which, together with the socioeconomic status and severity of the disease, precipitates states of depression and anxiety [4,11,19].

We observed that the sociodemographic characteristics of our population favor the development of psychiatric symptoms because of an organic illness, producing a sustained adrenergic response due to the permanent increase in stress caused by the inability to maintain oneself and pay for medical care [12].

The prevalence of psychiatric disorders in general hospitals is high and ranges from 25% to 65%. Anxiety and depression are the main complaints among patients admitted to hospitals with an organic disease [7,12]. Of the patients who developed symptoms of anxiety and depression, 40%-50% were unemployed at the time of the study. Anxiety symptoms were documented in 54.3%, and stress was related to lost working days and the possibility of dismissal due to absenteeism. In addition, patients with no job showed more symptoms of depression (52.2%) [8].

Patients with at least one comorbidity presented anxiety (53.9%) and depression (52.1%) in both measurements. The most frequent comorbidity was diabetes mellitus, followed by SAH, which increases the risk of developing psychiatric symptoms during the hospital stay [4,11,20].

In our study, people with chronic diseases had a 1.75 and 1.84 higher frequency of anxiety symptoms for both measures, respectively, than those with infectious diseases. Studies report that 19% of patients with pulmonary neoplasms and chronic diseases present depression and suicidal ideation related to physical pain; however, in our study, the presence of cancer did not turn out to be statistically significant for the presence of anxiety [4,10,12].

Anxiety is more prevalent in people over 40 years of age. In our series, the male gender developed more symptoms, and the mean age was 50 years [4]. Asthma, COPD, and OSA had common dyspnea, supplemental oxygen requirements, and prolonged hospital stays. These diseases precipitate excessive sympathetic activation, altering the systemic inflammatory response and disrupting the hypothalamic-pituitary-adrenal axis, producing a self-perpetuating cycle between mental disease and organic disease [12]. In addition to common symptoms, treatment for some lung diseases also represents a challenge since, in some cases, it consists of medications such as β-agonists, which are related to tachycardia, which is also a symptom of anxiety and predisposes to an increase in other psychological manifestations [9].

We found that 74.4% of the total sample presented anxiety symptoms, aggravating the clinical course of the disease. Patients with neoplastic diseases presented more depressive symptoms. On the other hand, acute respiratory diseases had higher anxiety symptoms in the first measurement but were not maintained in the second measurement, which may be due to an adaptive mechanism [3,4,11,12,21-24].

Among patients with lung cancer, symptoms of depression are common in up to 38% of cases. The risk factors are being young, being female, having a low income, and smoking. In our series, depressive symptoms increased from 58% to 63% between measurements, related to increased dyspnea and chronic pain [10-12,14,19,21].

Asthma was the chronic disease with the most anxiety symptoms in both measurements and even in the mixed pattern, with 86%, 86%, and 43%, similar to those reported worldwide [4].

The Hospital Anxiety and Depression Scale (HADS), which assesses emotional distress in patients with different conditions, has been applied to Hispanic patients, proving useful in detecting psychological disorders. It assesses cognitive and behavioral symptoms of anxiety and depression, such as insomnia, fatigue, and weight or appetite loss or gain.

This scale has several advantages, such as its simplicity and brevity, which facilitate its acceptance and use in clinical settings. In addition, it excludes elements of a somatic nature that could cause confusion in patients with associated physical symptoms; it has become a standard tool used in clinical and research settings, with a significant citation rate that supports its popularity and usefulness in diverse populations.

The period in which it was carried out, corresponding to the last seven days, has a sensitivity and specificity greater than 0.80. Our results showed that its application as a screening tool for respiratory diseases helps evaluate hospitalized patients. It is easy to apply and understandable for patients and healthcare personnel [15,16].

Among the causes of the underdiagnosis of psychiatric disorders in hospitalized patients are the presence of symptoms shared between certain diseases and depression, the effect of certain medications, and the difficulty of healthcare personnel to recognize the symptoms of depression and anxiety in the hospital context [9].

The evaluation of the patients was carried out during the COVID-19 pandemic, emphasizing the impact generated by the interruption of mental health services for outpatients, affecting their progress and treatment, and contributing to the increase in mental health problems [7].

Even though it is expected that symptoms of anxiety and depression will increase during the hospital stay, in the literature, no study reports subsequent evaluations, compared to our study where two measurements were applied, comparing the results of the second measurement with respect to the first one, guiding us in the decisions to make regarding medical management.

Although initially a patient could be classified in the category of symptoms without clinical relevance in the first measurement, as the days go by, these symptoms could increase requiring attention and hence the importance of performing more than one measurement; consequently, we consider it important to perform more of the measurement especially in patients with a prolonged hospital stay in order to evaluate the response to the management of anxiety and depression symptoms by mental health personnel.

This study has certain limitations since many patients had exacerbations of respiratory symptoms, which made it challenging to complete the questionnaires. Also, the sample size is limited. Other risk factors associated with anxiety and depression, such as low educational levels and drug and alcohol abuse, have not been evaluated.

## Conclusions

This study highlights the relevance of the symptoms of anxiety and depression among patients hospitalized due to respiratory diseases. The shared symptoms in these pathologies can make diagnosis difficult, which makes them underdiagnosed. Systematic screening and adequate evaluation not only of respiratory symptoms but also of psychological symptoms should be part of the diagnosis and make us able to identify patients who will benefit from multidisciplinary treatment.

The early detection and timely treatment of symptoms of anxiety and depression not only benefit the individual in terms of mental health but also have positive implications for the economic burden of the healthcare system. Integrating the routine assessment of these symptoms in healthcare can be crucial for achieving holistic management and improving outcomes at both individual and community levels. Therefore, we urge medical personnel not to underestimate the importance of these mental disorders and to take part in their care.
